# Associations of maternal pre-pregnancy body mass index and gestational weight gain with birth outcomes in Shanghai, China

**DOI:** 10.1038/srep41073

**Published:** 2017-01-25

**Authors:** Lingli Xiao, Guodong Ding, Angela Vinturache, Jian Xu, Yifang Ding, Jialin Guo, Liping Huang, Xuelei Yin, Jing Qiao, Inesh Thureraja, Xiaoming Ben

**Affiliations:** 1 Department of Neonatology, Shanghai First Maternity and Infant Hospital, Tongji University School of Medicine, Shanghai 201204, China; 2 Department of Pediatrics, Shanghai East Hospital, Tongji University School of Medicine, Shanghai 200120, China; 3 Department of Obstetrics & Gynaecology, John Radcliffe Hospital, Oxford University Hospital NHS Foundation Trust, Headley Way, Oxford, OX3 9DU, UK; 4 Department of Pediatrics, Punan Hospital of Pudong New District, Shanghai 200125, China; 5 Bachelor of medicine and bachelor of surgery (MBBS) 2012, Tongji University School of Medicine, Shanghai 200092, China

## Abstract

Recent data suggests that abnormal maternal pre-pregnancy body mass index (BMI) or gestational weight gain (GWG) is associated with unfavorable delivery outcomes. However, limited clinical evidence is available to support this correlation in China. Participating 510 mother-infant pairs were recruited from the Shanghai First Maternity and Infant Hospital, China, between January 1^st^ and 30^th^ 2016. Maternal pre-pregnancy BMI was categorized according to the China’s classification and GWG according to the 2009 Institute of Medicine recommendations (IOM). Linear regression tested the associations between pre-pregnancy BMI or GWG and length of gestation, birthweight, length, and head circumference. Logistic regression assessed the associations between pre-pregnancy BMI or GWG and macrosomic, small- (SGA) and large- (LGA) for-gestational-age infants. Overweight/obese women showed increased length of gestation and birthweight, but did not have a higher risk of macrosomic and LGA infants compared with normal weight women. Women with excessive GWG showed increased length of gestation, birthweight, length, and head circumference, and were more likely to deliver macrosomic and LGA infants compared with women with adequate GWG. Although a relatively low proportion of women from Shanghai area are overweight/obese or exhibit excessive GWG, both high pre-pregnancy BMI and excessive GWG influence perinatal outcomes.

Maternal nutritional status, reflected by pre-pregnancy body mass index (BMI) and gestational weight gain (GWG), is perceived as an important predictor of perinatal and even long-term outcomes for mothers and children. Accumulating evidence suggests that higher BMI prior to pregnancy is associated with an increased risk of preeclampsia, gestational diabetes, caesarean section, postpartum hemorrhage, and fetal macrosomia^1^,^2^,^3^,^4^, whereas maternal underweight is associated with an increased risk of preterm delivery and small-for-gestational-age (SGA)^5^,^6^. Maternal GWG has also been widely studied as an independent indicator of adverse pregnancy outcomes. Excessive GWG is associated with large-for-gestational-age (LGA), gestational diabetes, caesarean section, and postpartum weight retention^7^,^8^,^9^, while insufficient GWG is linked with low birthweight, preterm delivery, and SGA birth^10^,^11^. However, of the majority of the studies on the association between non-optimal maternal BMI or GWG and adverse pregnancy outcomes were conducted in developed countries of Europe and North America^12^,^13^,^14^. Furthermore, the extent that maternal BMI or GWG affects pregnancy outcomes varies among studies, among factors contributing to these differences are variations in the cut-offs used for BMI and GWG values, and characteristics of population studied such as demographics and ethnicity. For instance, women belonging to ethnic groups characterized by a small body size have been reported to gain less weight on average during pregnancy than larger women (Caucasians)^15^. In developing Asian countries, women generally have a lower BMI and a smaller GWG than those reported in developed countries from Europe and North America^16^. To date the information on the impact of maternal pre-pregnancy BMI and GWG is scarce, especially in developing Asian countries like China.

China, the world’s largest developing country, once regarded to have one of the leanest populations, is gradually catching up with western countries in terms of prevalence of overweight and obesity. The increases in the prevalence of overweight and obesity may be attributable to the China’s economic development, which has brought with it a high degree of westernization of the traditional Chinese diet and lifestyle^17^. The National Nutrition and Health Survey published in 2005 showed that, in China, the prevalence of overweight (BMI ≥ 24 kg/m^2^) in women 18–44 years of age increased from 16.8 to 21.8%, and the prevalence of obesity (BMI ≥ 28 kg/m^2^) increased from 3.1 to 6.1% from 1992 to 2002^18^. According to the 2009 Institute of Medicine (IOM) recommendations, more than half of childbearing-aged women living in north China exhibited excessive GWG^19^,^20^. The increasing prevalence of unhealthy preconception BMI and inappropriate GWG among women is of great concern for the Chinese public health community. Until now, however, there have been only a few reports evaluating the influence of maternal pre-pregnancy BMI and GWG on delivery outcomes in Chinese women^4^,^19^,^20^,^21^. In the present study, we aimed to investigate the current weight status of childbearing-aged women in Shanghai and to examine the possible relationships of non-optimal GWG and pre-pregnancy BMI with birth outcomes.

## Methods

### Study population

This retrospective cohort study was conducted in the Shanghai First Maternity and Infant Hospital, Tongji University School of Medicine, Shanghai, China. The hospital is one of the largest maternity hospitals with an annual delivery volume of approximately 25,000 babies, accounting for 12% of total number of deliveries in Shanghai. Eligibility criteria included: singleton pregnancy; age over 18 years old; residence in the metropolitan Shanghai area for at least 3 years prior to pregnancy; no report of assisted reproduction (spontaneous conception), pre-existing diabetes mellitus or gestational diabetes, chronic or pregnancy-associated hypertension, thyroid disorders, HIV infection or AIDS, and illicit drug use. Women were recruited to the study after delivery. From January 1^st^ to 30^th^ 2016, a total of 605 women met the eligibility criteria, among whom 519 women agreed to take part in this study (response rate 85.8%). Of these women, 9 women with missing values for major confounders were excluded. Therefore, 510 mothers were included in this study. Each woman participating in the study signed an informed consent form, and the research protocol for this study was approved by the Medical Ethics Committee of Shanghai First Maternity and Infant Hospital, Tongji University School of Medicine. All methods were performed in accordance with the relevant guidelines and regulations.

### Maternal interviews

Standardized face-to-face interviews were conducted with the women shortly after delivery by specially trained registered nurses in the hospital. The questionnaire collected information about: demographics (maternal age, education, marital status, and household income) and maternal characteristics (pre-pregnancy weight, and smoking and alcohol use during pregnancy). Other relevant information such as previous pregnancies and any medical conditions, current pregnancy complications, self-reported last menstrual period (LMP), and weight prior to delivery was obtained by interview and confirmed by maternal medical records. Clinical estimates of gestational age (ultrasound) were also obtained from maternal medical records. Gestational age was estimated based on the date of the self-reported LMP; if the LMP was unreliable or if there was a significant discordance between the clinical estimate and LMP (>2 weeks), the clinical estimation of gestational age was used^22^. Maternal height was measured during the interview. If the questionnaire was incomplete, after discharge, the mothers were contacted by telephone to obtain the missing information.

### Exposure information

The main exposures of interest were maternal pre-pregnancy BMI and GWG. At enrollment, from the maternal self-reported pre-pregnancy weight and measured height, we calculated pre-pregnancy BMI [weight (kg)/height (m)^2^]. In China, the two most commonly used BMI classifications for adults are the World Health Organization (WHO) standard and the recently developed Chinese BMI standard^23^. However, the Chinese standard that has lower cutoff points for BMI categories is more recommended in China because of recent evidence suggesting that Chinese people, as well as several other Asian Pacific populations, have an elevated risk for obesity-related diseases or conditions at a lower BMI than Caucasians^24^. Thus, the Chinese BMI classification standard was used in this study due to its better sensitivity and specificity for identifying risk factors such as hypertension, diabetes, and dyslipidemia in the Chinese population^23^. Therefore, women were divided into four groups based on pre-pregnancy BMI according to categories defined by the Working Group on Obesity in China as follows: underweight (BMI < 18.5 kg/m^2^), normal weight (18.5 kg/m^2^ ≤ BMI < 24.0 kg/m^2^), overweight (24.0 kg/m^2^ ≤ BMI < 28.0 kg/m^2^), and obese (BMI ≥ 28.0 kg/m^2^)^23^. Given the small number of women meeting the criteria for “obese” (n = 6), for meaningful comparisons this category was combined with the “overweight” category and collectively referred as overweight/obese. Statistical comparisons were carried out between three BMI groups: (1) “underweight”, (2) “normal weight”, and (3) “overweight/obese”. Women with normal BMI (18.5–24.0 kg/m^2^) were used as reference group for the analysis.

Maternal GWG was calculated as the difference between self-reported pre-pregnancy weight and the last clinically measured weight recorded prior to delivery. Since the Chinese Society of Obstetrics and Gynecology did not have an official guideline for weight gain during pregnancy, maternal GWG was categorized as inadequate, adequate, and excessive according to the 2009 Institute of Medicine (IOM) recommendations^25^. Inadequate GWG was defined as a weight gain during pregnancy of <12.5 kg in underweight women, <11.5 kg in normal weight women, <7 kg in overweight women, and <5 kg in obese women. Adequate GWG was defined as a weight gain during pregnancy of 12.5–18 kg in underweight women, 11.5–16 kg in normal weight women, 7–11.5 kg in overweight women, and 5–9 kg in obese women. Excessive GWG was defined as a weight gain during pregnancy of >18 kg in underweight women, >16 kg in normal weight women, >11.5 kg in overweight women, and >9 kg in obese women. Women with adequate GWG were used as reference group for the analysis.

### Outcome assessment

Neonatal outcomes included birthweight, length, head circumference, length of gestation, macrosomia, and SGA and LGA births. We were not able to analyze low birthweight (n = 5) and preterm delivery (n = 8) because of small number of infants who met these criteria. Infant sex, birth date, method of delivery, birthweight, crown-heel length, and head circumference were obtained from hospital delivery logs and medical records.

Low birthweight was defined as birthweight <2,500 g, whereas fetal macrosomia was defined as birthweight >4,000 g regardless of gestational age. A SGA infant was defined as an infant having a birthweight <10^th^ percentile, whereas a LGA infant was defined as an infant having a birthweight >90^th^ percentile for gestational age and sex according to a Chinese reference curve^26^. Preterm delivery was defined as birth at <37 completed weeks of gestation.

### Statistical analysis

Descriptive statistics were used to present the characteristics of the study population. Linear regression models were conducted to calculate beta (â) coefficients and 95% confidence intervals (CIs) evaluating the associations between exposures and length of gestation, birthweight, length, and head circumference (continuous variables). Logistic regression models were conducted to calculate odds ratios (ORs) and 95% CIs evaluating the associations between exposures and macrosomia, SGA and LGA births (dichotomous variables, yes/no).

We selected potential confounders for the multivariate linear or logistic models based on the associations reported in the literature and on the statistical considerations. We included in the final models as potential confounders those variables that were associated at p-values ≤ 0.2 with two or more birth outcomes in bivariate analysis. Confounders included were: maternal age, education, parity, smoking, alcohol use, infant sex, and household monthly salary. Multivariate models of birthweight, length, head circumference, macrosomia, SGA, and LGA were also adjusted for gestational age. Maternal pre-pregnancy BMI and GWG were mutually adjusted.

All results of regression models are expressed as comparisons with women with normal pre-pregnancy BMI or adequate GWG. Statistical analyses were carried out using SPSS software (SPSS Inc., Chicago, IL) based on two-tailed tests, and p < 0.05 indicated statistical significance.

## Results

Table 1 presents the socio-demographic characteristics of the study population. The mean (SD) pre-pregnancy BMI was 20.5 (2.6) kg/m^2^, and GWG was 15.1 (4.5) kg. With respect to pre-pregnancy BMI, 23.5% of the women were underweight, 65.9% were normal weight, and 10.6% were overweight/obese prior to pregnancy. Based on the IOM recommendations (2009), 20.6% of the women had inadequate GWG, 43.5% had adequate (normal) GWG, and 35.9% had excessive GWG. Maternal age ranged from 18 to 44 years old, and almost 80% of the women were between 25 and 34 years old. The vast majority (89.2%) of women had at least 13 years of education, three-quarters (75.3%) were primiparous, and two-thirds (65.5%) lived in households with a monthly income between RMB 10,000 and 30,000 yuan (per capita monthly income was 12,900 yuan in Shanghai reported in 2015). Nearly one-third (30.4%) of the women lived with a smoker during pregnancy or smoked themselves, although a few (6.3%) consumed alcohol regularly (at least once every two weeks).

Overall, 53.5% of the newborn infants were male. The mean (SD) birthweight, length, and head circumference were 3376.9 (447.7) g, 50.1 (0.7) cm, and 34.2 (1.1) cm, respectively. A total of 6.9% (n = 35) of infants were macrosomic, 10.6% (n = 54) were LGA, and 6.3% (n = 32) were SGA. However, very few infants were born of low birthweight (birthweight <2,500 g) (1.0%, n = 5) or preterm (1.6%, n = 8) (gestational age <37 weeks).

The unadjusted and adjusted linear regression results for the association of maternal pre-pregnancy BMI and GWG with birth outcomes are shown in Table 2. After adjusting for confounders, increased length of gestation (β = 0.40, 95% CI = 0.11 to 0.49) was found in overweight/obese women when compared with normal-weight women, but no associations were seen for underweight women. Similarly, women with excessive GWG also showed increased length of gestation (β = 0.23, 95% CI = 0.03 to 0.43) compared with women who had adequate GWG, but no associations were found in women who had inadequate GWG.

There were all significant positive associations of maternal pre-pregnancy BMI and GWG with birthweight (Table 2). Infants of overweight/obese women had increased birthweight (β = 144.8, 95% CI = 144.5 to 145.1), whereas infants of underweight women showed decreased birthweight (β = **−**104.2, 95% CI = **−**104.4 to **−**104.0), when compared with those of normal-weight women. Similarly, infants of women with excessive GWG showed increased birthweight (β = 216.3, 95% CI = 216.1 to 216.5), and infants with women with inadequate GWG showed decreased birthweight (β = **−**120.5, 95% CI = **−**120.8 to **−**120.3), when compared with those of women who had adequate GWG.

No significant associations were found between maternal pre-pregnancy BMI and birth length or head circumference. However, increased birth length (β = 0.29, 95% CI = 0.09 to 0.49) and head circumference (β = 0.33, 95% CI = 0.13 to 0.52) were observed in infants of women with excessive GWG when compared with women who had adequate GWG, but no associations were found in women with inadequate GWG (Table 2).

Because of the strong positive associations between pre-pregnancy BMI and GWG and birthweight found in the linear regression analyses, we further examined the potential relationships of pre-pregnancy BMI and GWG with macrosomia, SGA or LGA infants in logistic regression analysis. Table 3 summarizes the odds of macrosomia, SGA or LGA infants in women with non-optimal BMI or GWG. Overweight/obese women did not show an increase in the risk of macrosomia and LGA infants. Conversely, underweight women did not have an increased risk of SGA infants when compared with normal-weight women. However, women with excessive GWG exhibited increased risk of macrosomia (OR = 7.49, 95% CI = 2.42 to 23.19) and LGA infants (OR = 3.79, 95% CI = 1.82 to 7.90), whereas women with inadequate GWG exhibited increased risks of SGA infants (OR = 3.60, 95% CI = 1.57 to 8.30), when compared with women who had adequate GWG.

## Discussion

To the best of our knowledge, this is the first study from the metropolitan Shanghai area to assess birth outcomes with respect to pre-pregnancy BMI and GWG. The main findings of this study are that maternal excessive GWG but not overweight/obesity was associated with increased risk of macrosomic and LGA infants, whereas maternal inadequate GWG but not underweight was associated with an increased risk of SGA infants in a sample of women representative for the population of Shanghai. We also found that the proportion of underweight women prior to pregnancy was relatively high, whereas the proportion of women with excessive GWG was low.

### Weight status of childbearing-aged women in Shanghai

In the current study, we used the Chinese criteria instead of the WHO standard for BMI classification to categorize the women into different groups. Our preference is explained by the fact that: i) the WHO guidelines for BMI are based on data from Western countries, ii) increased awareness that, by the WHO guidelines, many Asian countries have a low prevalence of obesity, yet high rates of obesity-related diseases. The expert group of WHO recommended that individual countries should set their own criteria for BMI classification on the basis of their own morbidity and mortality data^27^. Therefore, more sensitive and specific criteria were developed for the Chinese women of childbearing age to estimate the risk of perinatal outcomes^23^. As shown in Table 4, we found that the proportion of underweight women in Shanghai (Southern China) (23.5%) was substantially higher than those reported from Tianjin (Northern China) (11.2%)^19^ and Western countries such as USA (5.5%)^28^ and Canada (3.9%)^29^, but was comparable to those reported from Jiangsu and Zhejiang provinces (nearby Shanghai) (22.7%)^21^ and Wuhan (Central China) (16.9%)^30^, and other Asian country such as Japan (14.8–20.0%)^31^,^32^,^33^. On the other hand, the proportion of women with excessive GWG in Shanghai (35.9%) was substantially lower than those reported from other domestic areas such as Tianjin (57.1%)^19^ and Wuhan (57.5%)^30^, and Western countries such as USA (57.9%)^28^ and Canada (57.7%)^29^, but higher than the reported proportions from Japan (7.1%-10.9%)^31^,^33^.

Several factors may explain the relatively thin body image of women observed in this study. Firstly, regional differences in dietary culture exist throughout China, particularly between southern and northern provinces due to climate and agriculture. Traditional Southern diet (e.g., Shanghai) is characterized by high intakes of rice and low intakes of wheat as staple foods. In contrast, Northern China (e.g., Tianjin) cultivates predominantly wheat, which is the staple food of the populations of this region^34^. Rice is a low-energy food that contains twice the amount of water and half of the energy compared with the same bulk of steamed bread (wheat)^35^. In addition, the colder climate in Northern China is associated with less physical activity and more energy-rich diets, which may increase body weight. These factors may explain, at least in part, higher prevalence of overweight and obesity in Northern than in Southern China. Secondly, women belonging to ethnic groups characterized by a small body size have been reported to gain less weight on average during pregnancy than larger women such as Caucasians^15^. Asian women generally have a lower BMI and a smaller GWG than those reported in North America^16^. However, a recent systematic review and meta-analysis found no significant association between dietary diversity and BMI status^36^. Furthermore, in the context of globalization, the Western fast food diet had become a major dietary pattern in modern China^37^. Some evidence also shows that the heterogeneity of food intake patterns is less often associated with BMI and obesity in women^38^. While differences in genetic makeup, environment, and behaviors across populations could explain discrepancies in maternal body weight in obstetric population from different reports, the knowledge regarding the seasonal food intake, dietary and activity patterns and obesity during pregnancy is still limited and require further interrogation.

### Association of pre-pregnancy BMI and GWG with birth outcomes

Both pre-pregnancy BMI and GWG are considered to be key determinants of infant health. Although these associations are extensively studied in the literature published from Western countries, much less is known about the association between BMI and GWG and birth outcomes in Chinese populations.

Only few studies examining the associations between maternal pre-pregnancy BMI and GWG and birth outcomes in Asian populations have been conducted, with inconsistent findings. For example, one study conducted in Nagasaki, Japan revealed that GWG below the IOM recommendation was associated with an increased risk of SGA births, while underweight showed no association with SGA births^39^. In contrast, another Japanese study from Oosaka, found that underweight was associated with an elevated risk of low birthweight infants, but inadequate GWG showed no association with low birthweight^32^. The same is true for studies of maternal pre-pregnancy BMI. Some studies reported that overweight/obese women had a higher incidence of fetal macrosomia and LGA infants^16^,^40^, or overweight/obese women were at increased risk of SGA infants^20^, while others reported that overweight/obesity was not associated with either low birthweight infants or fetal macrosomia^41^. However, two large Chinese studies conducted in Tianjin (n = 33,973) and Hebei, Jiangsu, and Zhejiang provinces (n = 292,568), respectively, showed that both overweight/obesity and excessive GWG increased the risks of fetal macrosomia and LGA infants, and both underweight and inadequate GWG were the risk factors for low birthweight and SGA infants^19^,^21^. A systematic review and meta-analysis indicates that the effect of pre-pregnancy BMI and GWG on delivery outcomes varies significantly among different groups and populations^42^. These differences may be attributed to population-specific genetic determinants, variable definitions used for of BMI and GWG categories, as well as differences in methodologies.

Our study has several limitations. Firstly, pre-pregnancy body weight was self-reported and not measured objectively. Overweight/obese women tend to underestimate their weight; it is possible that reporting bias occurred which may have influenced the grouping on women in the BMIs categories. Secondly, this study was based on data collected from a single hospital located in Shanghai area, which although reflects the characteristics of the pregnant population from this area, may affect the external validity of the findings. Thirdly, because the sample size was relatively small, we could not perform further subgroup analyses of all BMI categories. Finally, since there are no significant seasonal variations in the number of deliveries in our hospital and our population is representative for the obstetric population of the city of Shanghai, the study enrolled all women who delivered in one month of the year. However, we could not appreciate to what extent, if any, the seasonal variability had an impact on our findings. Although a relationship between seasons and the pattern of nutrition and physical activity may exist, the confounding effect of these factors on our results is unknown.

In conclusion, our study shows that association between adverse perinatal outcomes and non-optimal maternal nutrition exists in an urban Chinese population, supporting the findings from the Western populations. Also, our study draw attention to increasing rates of non-optimal maternal nutrition and rising incidence on obesity in Chinese women of childbearing age. Unlike other potential causes of poor birth outcomes, obesity is a preventable factor. Non-optimal GWG and pre-pregnancy BMI may be prevented with increased awareness and appropriate guidance. There is a misconception in the Chinese culture that increased GWG can be beneficial for the offspring’s health and growth. Pregnant women, especially those that are already overweight/obese, should be counseled about the importance of appropriate GWG which represents an underutilized opportunity for a public health intervention to prevent adverse perinatal outcomes. Similarly, underweight and overweight/obese women should also be counseled on the risk of poor delivery outcomes and advised on the importance of attaining optimal pre-pregnancy BMI when planning for pregnancy.

## Additional Information

**How to cite this article**: Xiao, L. *et al*. Associations of maternal pre-pregnancy body mass index and gestational weight gain with birth outcomes in Shanghai, China. *Sci. Rep.* **7**, 41073; doi: 10.1038/srep41073 (2017).

**Publisher's note:** Springer Nature remains neutral with regard to jurisdictional claims in published maps and institutional affiliations.

## Figures and Tables

**Table 1:** Maternal and infant’s characteristics according to pre-pregnancy body mass index (BMI) and gestational weight gain (GWG) (n = 510).

	Total sample (n = 510)	Pre-pregnancy BMI category			Gestational weight gain category		
		Underweight (n = 120)	Normal weight (n = 336)	Overweight and obese (n = 54)	Inadequate (n = 105)	Adequate (n = 222)	Excessive (n = 183)
**Maternal characteristic**							
Age (years)							
<25	23 (4.5%)	4 (3.3%)	17 (5.1%)	2 (3.7%)	5 (4.8%)	9 (4.1%)	9 (4.9%)
25–29	199 (39.0%)	63 (52.5%)	116 (34.5%)	20 (37.0%)	35 (33.3%)	89 (40.1%)	75 (41.0%)
30–34	206 (40.4%)	41 (34.2%)	144 (42.9%)	21 (38.9%)	50 (47.6%)	86 (38.7%)	70 (38.3%)
≥35	82 (16.1%)	12 (10.0%)	59 (17.6%)	11 (20.4%)	15 (14.3%)	38 (17.1%)	29 (15.8%)
Education (years)							
≤12 (High school)	55 (10.8%)	12 (10.0%)	33 (9.8%)	10 (18.5%)	5 (4.8%)	19 (8.6%)	31 (16.9%)
13–16 (Undergraduate school)	349 (68.4%)	82 (68.3%)	230 (68.5%)	37 (68.5%)	62 (59.0%)	163 (73.4%)	124 (67.8%)
≥17 (Postgraduate school)	106 (20.8%)	26 (21.7%)	73 (21.7%)	7 (13.0%)	38 (36.2%)	40 (18.0%)	28 (15.3%)
Parity							
0 (Primiparous)	384 (75.3%)	99 (82.5%)	241 (71.7%)	44 (81.5%)	80 (76.2%)	163 (73.4%)	141 (77.0%)
≥1 (Multiparous)	126 (24.7%)	21 (17.5%)	95 (28.3%)	10 (18.5%)	25 (23.8%)	59 (26.6%)	42 (23.0%)
Household monthly salary (¥)							
<10000	85 (16.7%)	22 (18.3%)	51 (15.2%)	12 (22.2%)	17 (16.2%)	33 (14.9%)	35 (19.1%)
10000–20000	196 (38.4%)	50 (41.7%)	126 (37.5%)	20 (37.0%)	36 (34.3%)	82 (36.9%)	78 (42.6%)
20001–30000	138 (27.1%)	30 (25.0%)	95 (28.3%)	13 (24.1%)	31 (29.5%)	64 (28.8%)	43 (23.5%)
>30000	91 (17.8%)	18 (15.0%)	64 (19.0%)	9 (16.7%)	21 (20.0%)	43 (19.4%)	27 (14.8%)
Smoking during pregnancy							
Yes/Lived with smoker	155 (30.4%)	35 (29.2%)	106 (31.5%)	14 (25.9%)	76 (72.4%)	152 (68.5%)	127 (69.4%)
No	355 (69.6%)	85 (70.8%)	230 (68.5%)	40 (74.1%)	29 (27.6%)	70 (31.5%)	56 (30.6%)
Alcohol use during pregnancy							
Yes	32 (6.3%)	9 (7.5%)	18 (5.4%)	5 (9.3%)	7 (6.7%)	12 (5.4%)	13 (7.1%)
No	478 (93.7%)	111 (92.5%)	318 (94.6%)	49 (90.7%)	98 (93.3%)	210 (94.6%)	170 (92.9%)
**Infant characteristic**							
Sex							
Male	273 (53.5%)	62 (51.7%)	186 (55.4%)	25 (46.3%)	65 (61.9%)	103 (46.4%)	105 (57.4%)
Female	237 (46.5%)	58 (48.3%)	150 (44.6%)	29 (53.7%)	40 (38.1%)	119 (53.6%)	78 (42.6%)
Gestational age (weeks)							
<37	8 (1.6%)	3 (2.5%)	5 (1.5%)	0 (0.0%)	3 (2.9%)	4 (1.8%)	1 (0.5%)
≥37	502 (98.4%)	117 (97.5%)	331 (98.5%)	54 (100.0%)	102 (97.1%)	218 (98.2%)	182 (99.5%)
Birthweight (g)							
<2500	5 (1.0%)	2 (1.7%)	3 (0.9%)	0 (0.0%)	2 (1.9%)	1 (0.5%)	2 (1.1%)
2500–4000	470 (92.2%)	115 (95.8%)	307 (91.4%)	48 (88.9%)	97 (92.4%)	217 (97.7%)	156 (85.2%)
≥4000	35 (6.9%)	3 (2.5%)	26 (7.7%)	6 (11.1%)	6 (5.7%)	4 (1.8%)	25 (13.7%)
Fetal growth							
SGA	32 (6.3%)	8 (6.7%)	23 (6.8%)	1 (1.9%)	15 (14.3%)	14 (6.3%)	3 (1.6%)
AGA	424 (83.1%)	107 (89.2%)	272 (81.0%)	45 (83.3%)	83 (79.0%)	197 (88.7%)	144 (78.7%)
LGA	54 (10.6%)	5 (4.2%)	41 (12.2%)	8 (14.8%)	7 (6.7%)	11 (5.0%)	36 (19.7%)
Birthweight (g) (mean ± SD)	3376.9 ± 447.7	3268.2 ± 3367.9	3390.2 ± 465.0	3535.9 ± 447.8	3180.3 ± 397.8	3302.9 ± 365.5	3579.5 ± 487.4
Birth length (cm)	50.1 ± 0.7	50.0 ± 0.4	50.1 ± 0.7	50.2 ± 0.9	50.0 ± 0.6	50.0 ± 0.5	50.3 ± 0.9
Head circumference (cm)	34.2 ± 1.1	34.1 ± 0.9	34.2 ± 1.1	34.4 ± 1.4	34.0 ± 1.1	34.1 ± 1.0	34.5 ± 1.1

**Table 2:** Associations (95% CIs) of pre-pregnancy body mass index (BMI) and gestational weight gain (GWG) with birth outcomes (n = 510).

Maternal weight status (No.)	Birth outcomes							
	Length of gestation (weeks)		Birthweight (g)		Length (cm)		Head circumference (cm)	
	Crude	Adjusted^b^	Crude	Adjusted^c^	Crude	Adjusted^c^	Crude	Adjusted^c^
	β (95% CI)	β (95% CI)	β (95% CI)	β (95% CI)	β (95% CI)	β (95% CI)	β (95% CI)	β (95% CI)
Pre-pregnancy BMI								
Underweight (120)	0.02 (−0.19, 0.23)	−0.03 (−0.24, 0.18)	−**122.1 (**−**122.3,** −121.9)**	−**104.2 (**−**104.4** −104.0)**	−0.15 (−0.36, 0.06)	−0.14 (−0.35, 0.08)	−0.18 (−0.39, 0.02)	−0.16 (−0.37, 0.06)
Normal weight (336)	Referent	Referent	Referent	Referent	Referent	Referent	Referent	Referent
Overweight/Obese (54)	0.37 (0.08, 0.66)**	0.40 (0.11, 0.69)**	145.7 (145.4, 145.9)**	144.8 (144.5, 145.1)**	0.12 (−0.17, 0.41)	0.09 (−0.21, 0.38)	0.21 (−0.08, 0.49)	0.23 (−0.07, 0.52)
GWG by the 2009 IOM recommendations^a^								
Inadequate (105)	−0.13 (−0.36, 0.10)	−0.14 (−0.37, 0.10)	−122.6 (−122.9, −122.4)**	−120.5 (−120.8, −120.3)**	0.07 (−0.17, 0.30)	0.08 (−0.15, 0.32)	−0.05 (−0.29, 0.18)	−0.07 (−0.31, 0.17)
Adequate (222)	Referent	Referent	Referent	Referent	Referent	Referent	Referent	Referent
Excessive (183)	0.26 (0.07, 0.46)**	0.23 (0.03, 0.43)*	276.5 (276.3, 276.7)**	216.3 (216.1, 216.5)**	0.35 (0.16, 0.55)**	0.29 (0.09, 0.49)**	0.42 (0.23, 0.62)**	0.33 (0.13, 0.52)**

^a^GWG within the IOM recommendations were 12.5–18, 11.5–16, 7–11.5, and 5–9 kg for underweight, normal weight, overweight, and obese women, respectively.

^b^Pre-pregnancy BMI and GWG were mutually adjusted and also adjusted for maternal age, education, parity, smoking, alcohol use, infant sex, and household monthly salary.

^c^Pre-pregnancy BMI and GWG were mutually adjusted and also adjusted for maternal age, education, parity, smoking, alcohol use, infant sex, household monthly salary, and gestational age.

*p < 0.05; **p < 0.01.

**Table 3:** Odds ratios (ORs) for birthweight according to pre-pregnancy body mass index (BMI) and gestational weight gain (GWG).

Maternal weight status	Birth outcomes								
	High birthweight (Macrosomia) n = 505 with 35 case			Small for gestational age (SGA) n = 456 with 32 case			Large for gestational age (LGA) n = 478 with 54 case		
	n	Crude OR (95% CI)	Adjusted OR (95% CI)^b^	n	Crude OR (95% CI)	Adjusted OR (95% CI)^b^	n	Crude OR (95% CI)	Adjusted OR (95% CI)^b^
Pre-pregnancy BMI									
Underweight	118 (3)	0.31 (0.09, 1.04)	0.34 (0.10, 1.22)	115 (8)	0.88 (0.38, 2.04)	0.94 (0.40, 2.23)	112 (5)	0.31 (0.12, 0.81)*	0.32 (0.12, 0.86)*
Normal weight	333 (26)	Referent	Referent	295 (23)	Referent	Referent	313 (41)	Referent	Referent
Overweight/Obese	54 (6)	1.48 (0.58, 3.78)	1.28 (0.44, 3.75)	46 (1)	0.26 (0.04, 2.00)	0.17 (0.02, 1.34)	53 (8)	1.18 (0.52, 2.68)	1.04 (0.42, 2.56)
GWG by the 2009 IOM recommendations^a^									
Inadequate	103 (6)	3.36 (0.93, 12.16)	4.28 (0.95, 17.03)	98 (15)	2.54 (1.18, 5.50)*	3.60 (1.57, 8.30)**	90 (7)	1.51 (0.57, 4.03)	1.76 (0.64, 4.85)
Adequate	221 (4)	Referent	Referent	211 (14)	Referent	Referent	208 (11)	Referent	Referent
Excessive	181 (25)	8.69 (2.97, 25.48)**	7.49 (2.42, 23.19)**	147 (3)	0.29 (0.08, 1.04)	0.28 (0.08, 1.02)	180 (36)	4.48 (2.20, 9.09)**	3.79 (1.82, 7.90)**

^a^GWG within the IOM recommendations were 12.5–18, 11.5–16, 7–11.5, and 5–9 kg for underweight, normal weight, overweight, and obese women, respectively.

^b^Pre-pregnancy BMI and GWG were mutually adjusted and also adjusted for maternal age, education, parity, smoking, alcohol use, infant sex, household monthly salary, and gestational age.

*p < 0.05; **p < 0.01.

**Table 4:** Between-study comparison of pre-pregnancy body mass index (BMI) and gestational weight gain (GWG) in childbearing-aged women.

Author/year	Country/city	Sample size	Pre-pregnancy BMI (mean ± SD)			GWG (mean ± SD)※		
			Underweight (%)	Normal weight (%)	Overweight/Obese (%)	Inadequate (%)	Adequate (%)	Excessive (%)
Yang *et al*.§ 2011–2013^30^	Wuhan, China	85,765	20.4 ± 2.3 kg/m^2^			17.4 ± 7.2 kg		
			16.9%	76.4%	6.7%	17.5%	25.0%	57.5%
Li *et al*.§ 2009–2011^19^	Tianjin, China	33,973	NA			NA		
			11.2%	64.6%	24.2%	9.8%	33.0%	57.1%
Liu *et al*.* 1993–2005^21^	Jiangsu and Zhejiang, China	273,365	NA			NA		
			22.7%	75.5%	1.9%	NA	NA	NA
The present study§ 2016	Shanghai, China	510	20.5 ± 2.6 kg/m^2^			15.1 ± 4.5 kg		
			23.5%	65.9%	10.6%	20.6%	43.5%	35.9%
Tanaka *et al*.# 2010–2013^31^	Osaka, Japan	1883	20.7 ± 2.9 kg/m^2^			10.3 ± 3.7 kg		
			20.0%	63.9%	16.1%	57.9%	31.2%	10.9%
Murakami *et al*.#,^32^	Sakata, Japan	633	20.9 ± 2.8 kg/m^2^			10.5 ± 3.4 kg		
			14.8%	77.2%	8.0%	NA	NA	NA
Enomoto *et al*.* 2013^33^	Yokohama, Japan	97,157	NA			NA		
			18.2%	71.1%	10.6%	63.8%	29.1%	7.1%
Deierlein *et al*.* 2001–2005^28^	the Pregnancy Infection and Nutrition (PIN) study, USA	363	24.2 ± 5.6 kg/m^2^			16.0 ± 5.4 kg		
			5.5%	65.4%	29.1%	12.7%	29.4%	57.9%
Ferraro *et al*.* 2002–2009^29^	the Ottawa and Kingston (OaK) Birth Cohort, Canada	4,321	25 ± 5.6 kg/m^2^			16.1 ± 6.8 kg		
			3.9%	56.2%	39.9%	13.0%	29.3%	57.7%

NA, not applicable.

^※^GWG within the IOM recommendations were 12.5–18, 11.5–16, 7–11.5, and 5–9 kg for underweight, normal weight, overweight, and obese women, respectively.

^§^Women were categorized as underweight (<18.5 kg/m^2^), normal weight (18.5–23.9 kg/m^2^), overweight (24.0–27.9 kg/m^2^), or obese (≥28.0 kg/m^2^) according to the standard of Working Group on Obesity in China.

^*^Women were categorized as underweight (<18.5 kg/m^2^), normal weight (18.5–24.9 kg/m^2^), overweight (25.0–29.9 kg/m^2^), or obese (≥30.0 kg/m^2^) according to the WHO criteria.

^#^Women were categorized as underweight (<18.5 kg/m^2^), normal weight (18.5–22.9 kg/m^2^), overweight (23.0–24.9 kg/m^2^), or obese (≥25.0 kg/m^2^) according to the Japan Society for Study of Obesity.
